# The importance of choosing the right strategy to treat small cell carcinoma of the cervix: a comparative analysis of treatments

**DOI:** 10.1186/s12885-021-08772-x

**Published:** 2021-09-23

**Authors:** Mariko Kawamura, Yutaro Koide, Taro Murai, Shunichi Ishihara, Yuuki Takase, Takayuki Murao, Dai Okazaki, Takahiro Yamaguchi, Kaoru Uchiyama, Yoshiyuki Itoh, Takeshi Kodaira, Yuta Shibamoto, Mika Mizuno, Fumitaka Kikkawa, Shinji Naganawa

**Affiliations:** 1 Department of Radiology, Nagoya University Graduate School of Medicine, 65 Tsurumai-cho, Shouwa-ku, Nagoya, Aichi 466-8550 Japan; 2 Department of Radiation Oncology, Aichi Cancer Center Hospital, Nagoya, Japan; 3 Department of Radiology, Nagoya City University Graduate School of Medical Sciences, Nagoya, Japan; 4 Department of Radiology, Toyohashi Municipal hospital, Toyohashi, Japan; 5 Department of Radiology, Japanese Red Cross Nagoya Daiichi Hospital, Nagoya, Japan; 6 Department of Radiology, Ichinomiya Municipal Hospital, Ichinomiya, Japan; 7 Department of Radiology, Okazaki City Hospital, Okazaki, Japan; 8 Department of Radiology, Gifu University Hospital, Gifu, Japan; 9 Department of Radiology, Kariya-Toyota General Hospital, Kariya, Japan; 10 Department of Obstetrics and Gynecology, Aichi Cancer Center Hospital, Nagoya, Japan; 11 Department of Obstetrics and Gynecology, Nagoya University Graduate School of Medicine, Nagoya, Japan

**Keywords:** Small cell carcinoma, Cervical cancer, Rare cancer, Chemotherapy

## Abstract

**Background:**

Standard treatments for small cell carcinoma of the cervix (SCCC) have not been established. In this study, we aimed to estimate the optimal treatment strategy for SCCC.

**Methods:**

This was a multicenter retrospective study. Medical records of patients with pathologically proven SCCC treated between 2003 and 2016 were retrospectively analyzed. Overall survival (OS) was plotted using the Kaplan-Meier method. Log-rank tests and Cox regression analysis were used to assess the differences in survival according to stage, treatment strategy, and chemotherapy regimen.

**Results:**

Data of 78 patients were collected, and after excluding patients without immunohistopathological staining, 65 patients were evaluated. The median age of the included patients was 47 (range: 24–83) years. The numbers of patients with International Federation of Gynecology and Obstetrics (FIGO) 2018 stages I-IIA, IIB-IVA, IVB were 23 (35%), 34 (52%), and 8 (12%), respectively. Of 53 patients who had undergone chemotherapy, 35 and 18 received SCCC and non-SCCC regimens as their first-line chemotherapy regimen, respectively. The 5-year OS for all patients was 49%, while for patients with FIGO stages I-IIA, IIB-IVA, IVB, it was 60, 50, and 0%, respectively. The 5-year OS rates for patients who underwent treatment with SCCC versus non-SCCC regimens were 59 and 13% (*p* < 0.01), respectively. This trend was pronounced in locally advanced stages. Multivariate analysis showed that FIGO IVB at initial diagnosis was a significant prognostic factor in all patients. Among the 53 patients who received chemotherapy, the SCCC regimen was associated with significantly better 5-year OS in both the uni- and multivariate analyses.

**Conclusion:**

Our results suggest that the application of an SCCC regimen such as EP or IP as first-line chemotherapy for patients with locally advanced SCCC may play a key role in OS. These findings need to be validated in future nationwide, prospective clinical studies.

**Supplementary Information:**

The online version contains supplementary material available at 10.1186/s12885-021-08772-x.

## Background

Small cell carcinoma of the cervix (SCCC) is a rare cancer. It comprises approximately 1–3% of all cervical neoplasms [1, 2]. Owing to its rarity, it is very difficult to plan a prospective study involving the affected patient population. Additionally, a standard treatment has not been established; therefore, debates regarding whether it should be treated with the same protocols used for localized SCCC or for advanced cervical cancer are ongoing. The Society of Gynecologic Oncology published a clinical document reviewing neuroendocrine tumors of the gynecologic tract in 2011 [3]. In this document, surgery is proposed as an optional therapy for early-stage SCCC, followed by adjuvant chemotherapy. Meanwhile, chemoradiotherapy is recommended for patients with advanced disease or those without indication for surgery, and chemoradiotherapy with etoposide/cisplatin (EP) combined with pelvic radiation is recommended based on data from multiple small retrospective cohort studies on prognostic factors of SCCC. Although multiple studies have recommended EP as the first-line chemotherapy regimen for SCCC, the number of patients who have been treated with EP in prior studies is very small. Since the EP regimen often causes severe pancytopenia, the use of EP combined with whole pelvic radiotherapy is not very common in Japan. However, after the publication of the document from the Society of Gynecologic Oncology in 2011, more institutions started to treat this disease as localized SCCC; however, the outcomes from this treatment approach still need to be evaluated., Furthermore, it has been reported that the irinotecan/cisplatin (IP) regimen for small cell lung cancer is as effective as EP [4], and since small cell carcinoma of the cervix and lungs have a similar protein expression [5], IP can be effective in both SCCC and small cell carcinoma of the lungs; however, the existing data are limited.

The purpose of this study was to evaluate the treatment strategy for SCCC by analyzing the data of patients who had undergone different treatments in nine major cancer care hospitals in the Tokai area of Japan. This study aimed to capture the existing SCCC treatment trends and to evaluate how treatment outcomes differed based on the treatment strategy, as well as the first-line chemotherapy regimen used.

## Methods

### Patient eligibility

Nine cancer treatment cooperation base hospitals certified by the Ministry of Health for providing high quality cancer care, located in the Tokai area of Japan, participated in this retrospective study. The institutional review board at each hospital approved participation in this multi-center study (research representative facility approval: Nagoya University Ethics Committee 2017–0010). The need for informed consent was waived due to the retrospective design. Patients were eligible when they (1) were pathologically diagnosed with SCCC and (2) received an initial diagnosis between 2003 and 2016. Patients with a history of chemotherapy or radiotherapy used to treat different malignant tumors and/or double primary malignant tumors were excluded. Data regarding patient diagnosis, treatment regimens, treatment-related toxicities, and treatment outcomes (overall survival, local control, distant control) were collected.

### Patient diagnosis

Histopathologic diagnosis was based on morphological criteria, and immunohistochemical staining was not a prerequisite at the time of case accumulation. However, considering that this was a retrospective study and that we did not perform a central pathological review, we decided to exclude patients without immunohistochemical information such as synaptophysin, chromogranin, and CD56 from further evaluation of survival. All tumors were staged clinically by the International Federation of Gynecology and Obstetrics (FIGO) 2009 [6] and radiologically by the Union for International Cancer Control ver. 7 (UICC) [7] which were then converted to FIGO 2018. When patients underwent upfront surgery, pathological staging was prioritized over radiological staging; however, when patients underwent neoadjuvant chemotherapy prior to surgery, radiological staging prior to chemotherapy was prioritized.

### Treatment regimen

For chemotherapy recipients, the chemotherapy regimen was classified as either an SCCC or a non-SCCC regimen. The patients were divided into two groups depending on the regimen received. The patients were assigned into an SCCC regimen group when they received either IP or EP as their first line chemotherapy. Changing CDDP to carboplatin because of kidney function impairment was allowed, and these patients were included in the SCCC regimen group. All other patients who had undergone chemotherapy with weekly CDDP (wCDDP) or CDDP with fluorouracil (5-FU) and other treatments commonly used in cervical cancer treatment were assigned to the non-SCCC regimen group. For those who had CCRT followed by chemotherapy, patients were grouped by the chemo regimen they received during CCRT. This was done due to difficulty in differentiating the reason for changing the following chemo regimen, as adjuvant or second-line due to progressive disease after CCRT. Depending on the treatment strategy, patients were grouped as follows: (1) surgery only; (2) local first: surgery followed by chemotherapy or chemoradiotherapy; (3) neoadjuvant chemotherapy or chemoradiotherapy (NAC) prior to surgery; (4) concurrent chemoradiotherapy (CCRT); and (5) chemotherapy only.

### Statistical analyses

Overall survival (OS) was plotted using the Kaplan-Meier method, and the log-rank test was used to assess differences in survival between pairs of groups. Multivariate analysis was performed using Cox regression analysis for variables that were significant in the univariate analysis. For all statistical tests, *p* < 0.02 was used to indicate significance. All statistical analyses were performed using EZR (Saitama Medical Center, Jichi Medical University, Saitama, Japan), a graphical user interface for R (The R Foundation for Statistical Computing, Vienna, Austria).

## Results

### Patient characteristics and treatment strategies

The data from 78 patients was collected. One patient who had not received any treatment and died a month after diagnosis was excluded. Of the 77 patients, immunohistopathological data were not available for 12, which left 65 patients for inclusion in the evaluation. The patient characteristics along with chemotherapy regimens and treatments used are shown in Table 1. The median age of the included patients was 47 (range: 24–83) years, and the numbers of patients with FIGO 2018 stages I-IIA, IIB-IVA, and IVB were 23 (35%), 34 (52%), and 8 (12%), respectively. The median size of the primary tumor was 38 (range: 3–117) mm. Thirty-four (52%) patients had lymph-node metastasis at diagnosis, and 53 (82%) had received chemotherapy. Of these 53 patients, 35 and 18 received SCCC and non-SCCC regimens as first-line chemotherapy, respectively. Regarding the treatment strategy, 12 patients had CCRT with SCCC regimens, all with concurrent EP and radiotherapy. Four had adjuvant therapy after surgery, four had NAC prior to surgery, and four had curative intent CCRT. However, in four curative intent CCRT patients, only two had brachytherapy after whole pelvic radiotherapy. Of the 18 patients who received non-SCCC regimens, 17 received non-SCCC regimens concurrently with radiotherapy and only one patient with stage IVB had non-SCCC chemotherapy alone.

**Table 1:** Patients characteristics

	ALL Patients	
	***N*** = 65	(%)
**AGE (range)**	47(24–83) y.o.	
**TUMOR SIZE**	38(3–117) mm	
**Performance Status**		
0–1	64	(98)
≧2	1	(2)
**FIGO (2018)**		
I-IIA	23	(35)
IIB-IVA	34	(52)
IVB	8	(12)
**LYMPH NODE METASTASIS**		
–	31	(48)
+	34	(52)
**CHEMOTHERAPY**		
**No chemo**	**12**	**(18)**
**Small cell regimen (SCC)**	**35**	**(54)**
*CDDP(or CBDCA) + VP16*	*22*	
≧4course	11	
≦3course	11	
*CDDP(or CBDCA) + CPT11*	*13*	
≧6course	10	
≦5course	3	
**Non-small cell regimen**	**18**	**(28)**
*CDDP(or CBDCA) + 5FU*	*8*	
≧3course	4	
≦2course	4	
*NED + 5FU*	5	
≧3course	3	
≦2course	2	
*wCDDP*	*3*	
*DOC or PTX + CBDCA*	*2*	
**TREATMENT STRATEGY**		
**Surgery**	**12**	**(18)**
**Local first**	**25**	**(38)**
Surgery+CCRT	10	
w/SCC regimen	4	
Surgery+Chemo	15	
w/SCC regimen	15	
**NAC**	**7**	**(11)**
CCRT+Surgery	4	
w/SCC regimen	4	
Chemo+Surgery	3	
w/SCC regimen	2	
**CCRT(w/BT**^a^**)**	**14(11)**	**(21(17))**
w/SCC regimen	4(2)	
**Chemo**	**7**	**(11)**
w/SCC regimen	**6**	

^a^w/BT; with brachytherapy, RT; radiotherapy

### Treatment outcome

#### Local control

Twelve patients experienced local recurrence or local residuals. Local recurrent rate along with treatment strategy and FIGO stages are summarized in Table 2. Local recurrence was not observed in patients who were treated with CCRT using the SCCC regimen in local first, NAC, or curative intent. Interestingly, FIGO stage I-IIA patients had the highest local failure rate, with 26% having failed locally. Moreover, local recurrence was more common in the surgery only group and the group that received surgery followed by a non-SCCC regimen as adjuvant therapy despite the fact that patients who had upfront surgery tended to have early stage disease.

**Table 2:** Application of chemotherapy, treatment strategy and their outcome by UICC and FIGO stages

		Treatment strategy											Local Failure (%)	Distant Failure (%)	5y-OS (%)
		Surgery only	Local first			NAC			CCRT		Chemo				
			SCCC		N-SCCC^b^	SCCC		N-SCCC^b^	SCCC	N-SCCC	SCCC	N-SCCC			
			C	CCRT		C	CCRT								
FIGO 2018	I-IIA	11	8	0	2	0	2	0	0	0	0	0	6(26)	8(34)	60%
	IIB-IVA	1	7	4	4	2	2	1	3	9	1	0	5(15)	16(47)	50%
	IVB	0	0	0	0	0	0	0	1	1	5	1	1(13)	NA	NA
Local Failure(%)		4(33)	3(23)	0(0)	2(33)	0(0)		0(0)	0(0)	2(20)	1(17)	0(0)			
Distant Failure(%)^a^		4(33)	7(41)	1(25)	6(100)	0(0)		1(100)	0(0)	4(57)	NA				
5y OS(%)		69	61		NA	100		NA	75	30	NA				

*SCCC* small cell carcinoma of cervix regimen, *N-SCCC* non-small cell carcinoma of cervix regimen, *NA* not applicable, *OS* overall survival, *C* chemotherapy, *CCRT* concurrent Chemoradiotherapy

^a^Patients with FIGO IVB at diagnosis were excluded from distant failure rate analysis

^b^All patients treated with N-SCCC regimen in local first or NAC had concurrent radiotherapy

#### Distant metastasis

The distant failure rate was higher in advanced stage patients. Regarding the treatment strategy, patients who received a non-SCCC regimen had a higher failure rate in local control as well as distant control (Table 2).

#### Survival

**Fig. 1:**
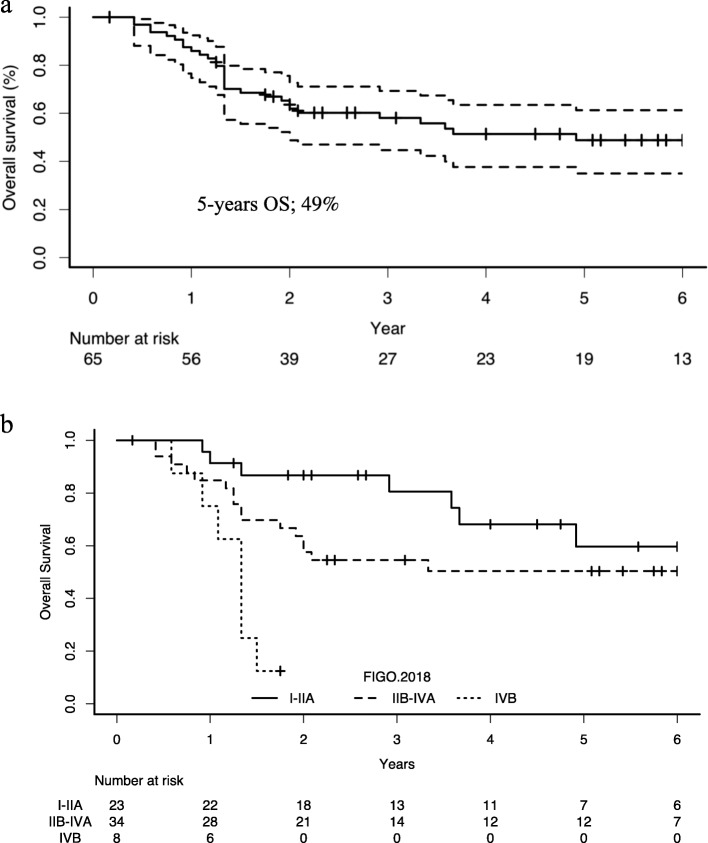
Overall survival of all patients and differences in survival depending on the FIGO 2018 staging. **a**. Overall survival of all patients with the coinciding 95% confidence intervals. Among patients who died from SCCC, the most common time of death was within 2 years of their diagnosis. **b**. Overall survival of patients as classified by FIGO I-IIA, IIB-IVA, and IB representing local, loco-regional, and systemic disease. Patients with FIGO IVB at diagnosis had the worst survival

The 5-year OS for all patients was 49% (Fig. 1a). The differences in survival depending on the FIGO stage are shown in Fig. 1b. The 5-year OS rate according to FIGO stages, and based on treatment strategy are also summarized in Table 2. The 5-year OS rates for FIGO stages I-IIA, IIB-IVA, and IVB was 60, 50, and 0%, respectively.

The results of the univariate and multivariate Cox regression analyses of all patient and patients who received chemotherapy for OS are summarized in Table 3. FIGO stage IVB at diagnosis was a significant poor prognostic factor in both the uni- and multivariate analyses of all 65 patients. Among the 53 patients who received chemotherapy, the use of the SCCC regimen showed significantly better 5-year OS in the uni- and multivariate analyses.

**Table 3:** Prognostic factors of overall survival derived from the univariate and multivariate Cox regression models for all patients and patients who recieved chemotherapy

Variable	All patients (n = 65)				Patients who received chemotherapy (*n* = 53)			
	Univariate analysis		Multivariate analysis		Univariate analysis		Multivariate analysis	
	HR(95%CI)	*p*	HR(95%CI)	*p*	HR(95%CI)	*p*	HR(95%CI)	*p*
With chemo	1.85(0.64–5.28)	*.25*						
Tumor size ≦4 cm (vs > 4 cm)	0.61(0.30–1.25)	*.18*			0.86(0.40–1.84)	*.69*		
Lymph node metastasis	2.26(1.08–4.74)	*.03*			1.86(0.81–4.28)	*0.14*		
FIGO IVB	4.47(1.79–11.20)	*<.01*	4.78(1.52–15.07)	*<.01*	4.11(1.60–10.57)	*<.01*	4.87(1.53–15.54)	*<.01*
With surgery	0.33(0.16–0.69)	*<.01*	0.72(0.28–1.85)	*.49*	0.37(0.17–0.80)	*.01*	0.74(0.29–1.89)	*.53*
With chemo (vs without)	3.75(1.22-				(vs SCC regimen)			
Non-SCC regimen	11.53)	*.02*	2.96(0.87–10.11)	*.08*	2.90(1.35–6.22)	*<.01*	3.50(1.53–8.03)	*<.01*
SCC regimen	1.21(0.39–3.71)	*0.74*			1		1	

*SCC* small cell carcinoma

**Fig. 2:**
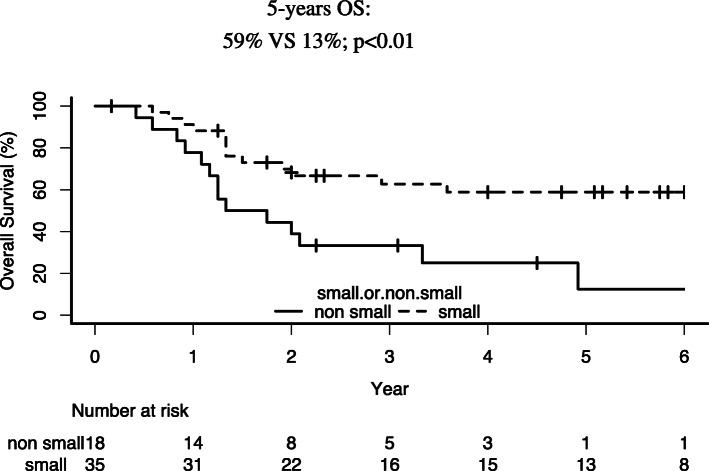
Differences in survival depending on the chemotherapy regimen used. Patients treated with the non-small chemotherapy regimen tended to have worse survival rates than those treated with the small cell carcinoma regimen

The 5-year OS rates for patients who received SCCC versus non-SCCC regimens were 59 and 13% (*p* < 0.01), respectively (Fig. 2). There was no difference between IP and EP (Supplemental data, Fig. B). A trend toward better OS in patients treated with the SCCC regimen was pronounced in locally advanced stages. (Supplemental data, Fig. A).

As in Table 2, the majority patients with FIGO stage I-IIA had surgery only or upfront surgery followed by chemotherapy. Among 11 patients who received surgery-only, three had died by the time of data collection. Additionally, two had died after 3 years of primary treatment. In the 10 patients who received local-first, only two patients had surgery followed by adjuvant CCRT with a non-SCCC chemotherapy regimen. One patient died just before the 5th year of follow-up, and one survived for at least 4 years (Supplemental data, Fig. A). Eight patients received adjuvant chemotherapy following surgery with an SCCC regimen, and in those eight patients, four died of metastases from SCCC. They all had EP without concurrent radiotherapy. Two patients who had CCRT prior to surgery (NAC) with SCCC regimen and both survived at the time of data recruitment.

For FIGO stage IIB-IVA patients, one patient had surgery only and died 5 months after the surgery. Of other 33 patients, patients 5 years OS of patients received SCCC and non SCCC regimen were 78 and 14%, respectively.

Fifteen patients had upfront surgery (local first) followed by adjuvant CCRT or chemotherapy. Of the 15 patients with upfront surgery, eleven patients received adjuvant therapy with an SCCC regimen: four had CCRT with an EP regimen and seven had chemotherapy with an EP (*n* = 4) or IP (*n* = 3) regimen. Four patients who had adjuvant therapy with a non-SCCC regimen were treated with concurrent radiotherapy, but all of them died from metastasis of SCCC. NAC was performed in five patients, one of whom received a non-SCCC regimen concurrently with radiotherapy who has died of SCCC after 15 month. The remaining four patients received a SCCC-regimen. Two were treated with CCRT with an EP regimen and the other two were treated with an IP regimen without radiotherapy. All four patients who received NAC with an SCCC regimen were alive at the time of data collection. CCRT was performed in twelve patients, and three patients received an SCCC regimen. All received concurrent EP and achieved complete remission at the time of data collection.

FIGO stage IVB patients, two had CCRT, one with SCCC regimen and one with a non-SCCC regimen, but both died of SCCC,

Fourteen patients received CCRT with curative intent. Ten patients received a non-SCCC regimen, and four received an SCCC regimen concurrently with radiotherapy. Among the four patients who received CCRT and a concurrent SCCC regimen, all received an EP regimen and were treated in the same hospital. The 5-year OS of patients who had SCCC-CCRT and non-SCCC-CCRT were 75 and 30%, respectively (Table 2, Supplemental data, Fig. C). Although there were only four patients who were treated with CCRT using an SCCC regimen, none had local recurrence. There were two patients who had CCRT with a non-SCCC regimen followed by IP. These patients were not included in the SCCC group because they had IP after CCRT since they could not achieve complete remission with CCRT. Therefore, we classified these two patients as having received IP as second-line treatment, not as an adjuvant treatment of CCRT.

When evaluating the primary treatment strategy for non-stage IVB patients, who were treated with SCCC regimen, the OS rates for the NAC, CCRT, and chemo were surperior compared to local first (Supplemental data, Fig. D).

#### Causes of death

Among all 31 patients who had died by the time of the analysis, death was attributed to SCCC metastases, and there were no treatment-related deaths. Nine had dissemination to the brain and/or meninges (Supplemental data, Table F). No patient underwent prophylactic cranial irradiation. Other metastatic sites included the lungs, liver, bones, and pleura/peritoneum.

## Discussion

We conducted a multicenter retrospective study to assess the current state of SCCC treatment and how different treatment methods affect the outcomes of this very rare but aggressive disease. We found that the treatment approach for this disease varied among institutions even though patients were recruited from governmentally certified cancer centers only.

There was a trend that patients were treated with non-SCCC regimens when chemotherapy was applied concurrently with radiotherapy. However, among patients who were treated with chemotherapy, application of SCCC regimens seemed to improve survival, especially among locally advanced SCCC patients.

Although the necessity for adjuvant chemotherapy for early SCCC remains unclear, it should be noted that local control of early stage was lower than that of advanced disease. As the 5-year OS for patients with early stage SCCC who received surgery only was superior to that of those who received adjuvant chemotherapy, there may be a group of patients who do not require adjuvant chemotherapy but at same time, there is a selection bias by clinicians. However, we hypothesize that there are certain patients who may benefit from chemotherapy, and those who benefit from chemotherapy may have better outcomes if chemotherapy or chemoradiotherapy is applied prior to surgery. Applying NAC to early stage patients may be excessive for some patients; however, further investigation is required to determine which patients with early stage SCCC will require chemotherapy.

Patients with FIGO IIB-IVA had 5-year OS of 50% in all and 78% in patients treated with SCCC regimen, which is superior to previously reported findings of earlier stage (Table 4) [8–11]. Therefore, we hypothesize that application of a SCCC chemotherapy regimen such as EP or IP may be more important for locally advanced SCCC. Wang et al [12] retrospectively analyzed the data of 179 patients with SCCC and found that patients treated with five cycles of EP had better outcomes. In this study, 22 patients were treated with the EP regimen and half received more than four cycles. Those who received more than four cycles of EP were more likely to have received NAC or CCRT rather than adjuvant treatment after surgery. Twelve patients received CCRT with the SCCC regimen, four as adjuvant therapy after surgery, four as NAC prior to surgery, and four as curative intent; all patients received EP. This was a retrospective study including a very small number of patients, but it should be noted that no patient who received CCRT with EP had local failure, and only one patient had distant metastasis. We believe that CCRT with concurrent EP is an effective treatment, especially among patients with locally advanced SCCC. Additionally, it may be beneficial in selected patients with early stage SCCC because although we failed to prove statistical significance owing to the small number of patients, the patients in the NAC group tended to have better 5-year OS than those in the local-first group, despite the fact that the NAC group included more patients with advanced stage disease. This may be because there is a bias in selecting good responders for chemotherapy, which may be a prognostic factor for OS. In contrast, this may be the key to the understanding of the poor OS identified among early stage patients. As previously described, certain early stage SCCC patients may not require chemotherapy; however, identification of patients who could benefit from chemotherapy in early stage and to whom chemotherapy could be administered prior to surgery may be helpful in improving their outcomes. No patients who received CCRT were treated with concurrent IP likely since diarrhea is a common major adverse effect of irinotecan and of whole pelvic irradiation. Patients who underwent upfront surgery followed by chemotherapy were likely to receive IP. Therefore, although there was no difference in OS between the patients who received IP or EP, considering that patients who underwent upfront surgery tended to have earlier-stage tumors, there may have been patient selection bias.

**Table 4:** Prognosis of SCCC in past studies and the present study

study	FIGO	n	survival	Treatment
Lee et al. (2008) [8]	IB-IIA	24	5y OS: 53%	Ope + chemo
	IB-IIA	24	5y OS:46%	Ope + CCRT
Kuji et al. (2013) [9]	IB-IIB	7	4y OS: 29%	Ope ± RT
	IB-IIB	21	4y OS: 65%	Ope ± CCRT or chemo
Zivanovic et al. (2009) [10]	IA2-IB2	6	3y OS: 83%	CCRT
Hoskins et al. (2003) [11]	I-IIA	16	3y PFS: 80%	CCRT±chemo
Present study	I-IIA (all)	23	5y OS: 60%	
	I-IIA (SCC)	10	5y OS: 53%	
	IIB-IVA (all)	34	5y OS: 50%	
	IIB-IVA (SCC)	19	5y OS: 78%	

*all* all patients, *SCCC* patients treated with SCCC regimen

The type of chemotherapy used differed greatly in each institution. Although our series recruited patients from nine institutions, two contributed more than 20 patients (high volume centers) and others less than 10 (low volume centers). The SCCC regimen was extensively used in these two high-volume centers; therefore, this too may be an inherent bias. Some retrospective studies have reported that the clinical stage at diagnosis and application of CDDP-based chemotherapy are prognostic factors for SCCC [12–15]. We also found that patients at stage IVB had significantly lower 5-year OS than the patients at other stages. However, those who had received non-SCCC chemotherapy also had CDDP in their regimen; therefore, we believe that the key drugs for treating SCCC are etoposide or irinotecan.

The role of surgery is not very clear even at the early stage. Ishikawa et al [16] identified the superiority of surgery over CCRT in early-stage SCCC in Japan, but they included only five patients treated with CCRT, and they did not report on the use of chemotherapy regimens for CCRT. Based on our results, most Japanese institutions are using CCRT with a cervical cancer regimen and not with an SCCC regimen. This strategy may have caused the poor CCRT outcomes because, based on our data, using CCRT with the SCCC regimen for locally advanced stage patients was not inferior to using the SCCC regimen with chemotherapy or CCRT as adjuvant systemic therapy for the local-first group (Supplemental data, Fig. E).

Due to the retrospective nature of this study, we may have underestimated the local control for patients with distant metastases. When a patient has distant metastasis to critical organs such as the lungs and liver, a comprehensive evaluation for local disease is not carefully performed unless they have some critical problem. Therefore, we may have underestimated the local control for advanced disease, especially among those who died shortly after their diagnosis. However, it should be noted that there was no local failure in the surviving patients who were treated with CCRT with the SCCC regimen.

SCCC is rare, but very aggressive, and the OS rate is usually very poor. Approximately half of the patients died from SCCC, and most died within 2 years of their diagnosis. Our sample included nine (14%) patients who died from dissemination to the brain and/or meninges. Once dissemination occurs, treatment is futile. For lung carcinoma, some reports have recommended prophylactic cranial irradiation; however, in our study, no patient received prophylactic cranial irradiation. Additionally, since half of the patients who had dissemination to the brain and/or meninges had stage IVB disease at diagnosis, it is difficult to decide whether to recommend prophylactic cranial irradiation (Supplemental data, Table E).

The major limitation of our study is that we did not perform a central pathological review. Although we only included patients from government-certified cancer care hospitals, we decided to evaluate the data for patients with immunohistochemical staining results available. Another limitation is that this was a retrospective study, and thus there may be inherent biases especially because of small number patients and wide variety in treatment strategies used. Therefore, our statistical assessment may lack credibility; therefore, we provided as much of the actual data as possible. Since SCCC is a rare cancer, performing a prospective study in one institution may be difficult. However, because it is rare, we should share these data in the hope of improving SCCC outcomes in the future.

## Conclusion

Our results suggest that the application of an SCCC regimen such as EP or IP as first-line chemotherapy for patients with locally advanced SCCC may play a key role in OS. These findings need to be validated in future nationwide, prospective clinical studies.

## Supplementary Information

**Additional file 1.**

## Data Availability

The authors confirm that the data supporting the findings of this study are available within the article and its supplementary materials. Furthermore, all data that support the findings of this study are available from the corresponding author, MK, upon reasonable request.

## Abbreviations

SCCC: Small cell carcinoma of the cervix
OS: Overall survival
UICC: Union for International Cancer Control
EP: Etoposide/cisplatin
FIGO: Federation of Gynecology and Obstetrics
CDDP: Cisplatin
CBDCA: Carboplatin
VP16: Etoposide
CPT11: Irinotecan
DOC: Docetaxel
PTX: Paclitaxel
RT: Radiotherapy
IP: Irinotecan/cisplatin
5-FU: Fluorouracil
NAC: Neoadjuvant chemotherapy or chemoradiotherapy
CCRT: Concurrent chemoradiotherapy
HR: Hazard ratio
