# Supplementary material for: The importance of choosing the right strategy to treat small cell carcinoma of the cervix: a comparative analysis of treatments
Source: BMC Cancer. 2021 Sep 23;21:1046. doi: 10.1186/s12885-021-08772-x (PMC8461987; doi:10.1186/s12885-021-08772-x)
Supplement: Supplementary file 1 — Additional file 1. [file 12885_2021_8772_MOESM1_ESM.pdf]

The importance of choosing the right strategy to treat small cell carcinoma of the cervix: a comparative analysis of treatments

Mariko Kawamura[1,] Yutaro Koide[2], Taro Murai[3], Shunichi Ishihara[4], Yuuki Takase[1,5], Takayuki Murao[6], Dai Okazaki[7], Takahiro Yamaguchi[8], Kaoru Uchiyama[9], Yoshiyuki Itoh[1], Takeshi Kodaira[2], Yuta Shibamoto[3], Mika Mizuno[10], Fumitaka Kikkawa[11], and Shinji Naganawa[1]

Figure A) Differences in survival according to the chemotherapy regimen in each FIGO stage

FIGO I-IIA

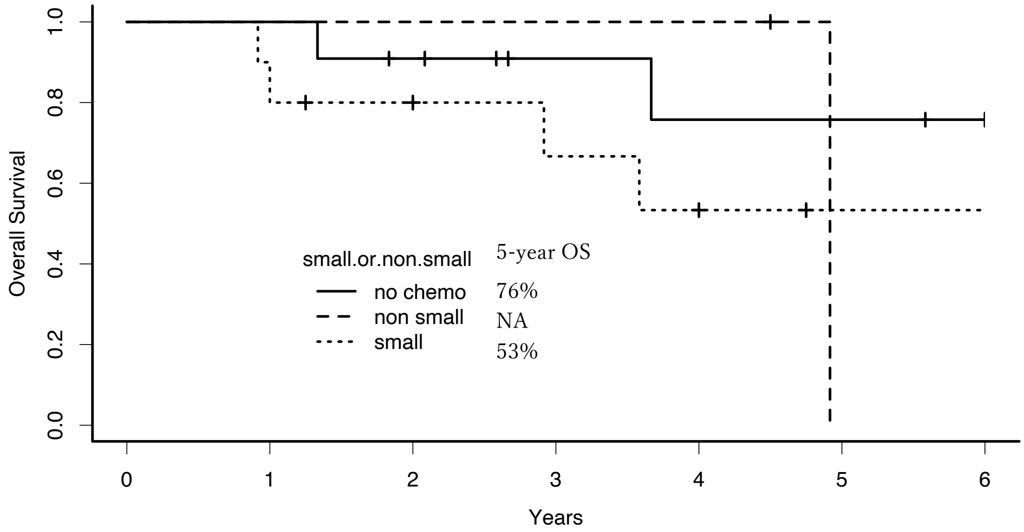

Number at risk

	0	1	2	3	4	5	6
no chemo	11	11	9	6	5	5	4
non small	2	2	2	2	2	0	0
small	10	9	7	5	4	2	2

FIGO IIB-IVA

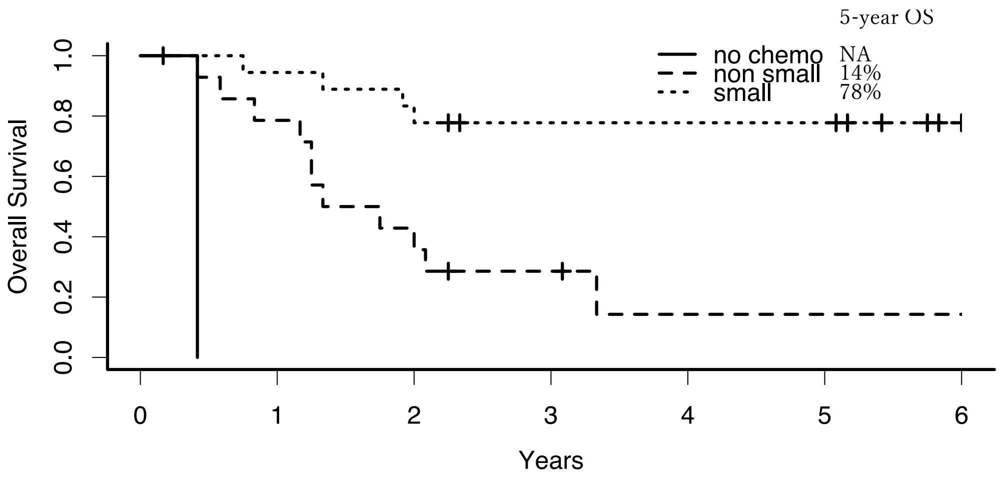

Number at risk

	0	1	2	3	4	5	6
no chemo	1	0	0	0	0	0	0
non small	14	11	6	3	1	1	1
small	19	17	15	11	11	11	6

FIGO IVB

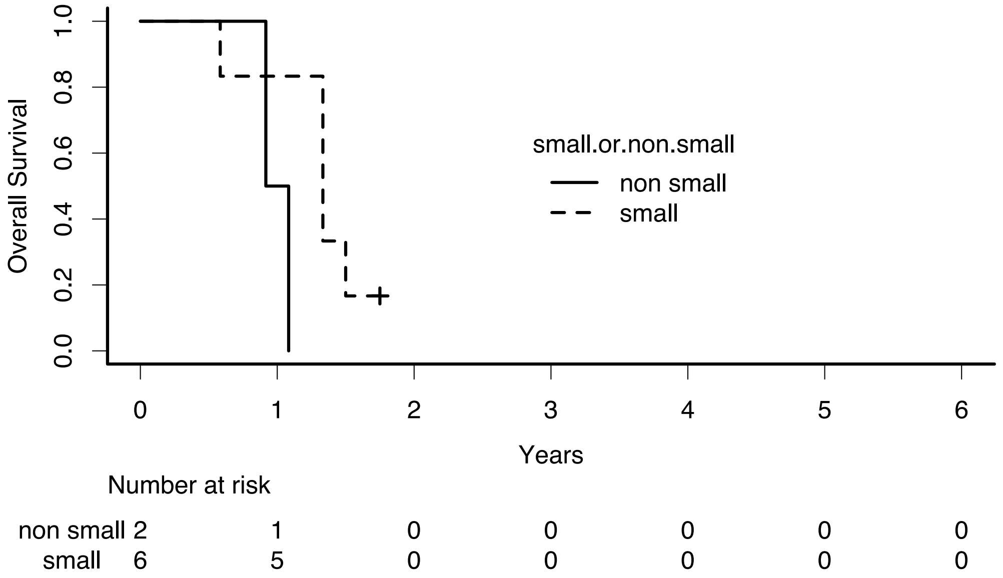

Figure B) Differences in survival with use of IP vs EP

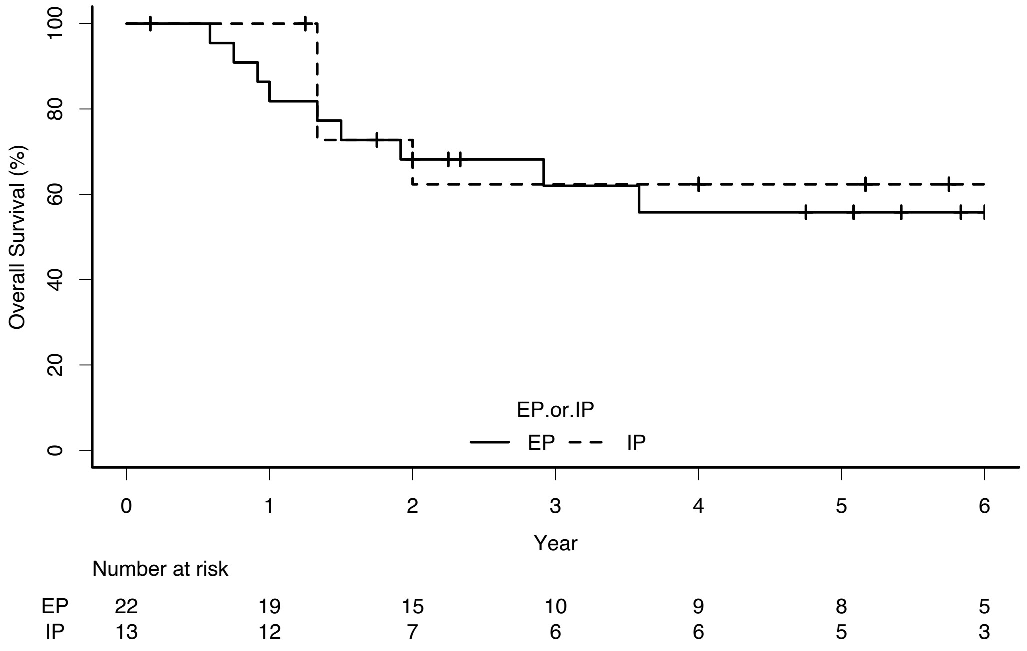

Figure C) Differences in survival according to the chemotherapy regimen for patients treated with CCRT

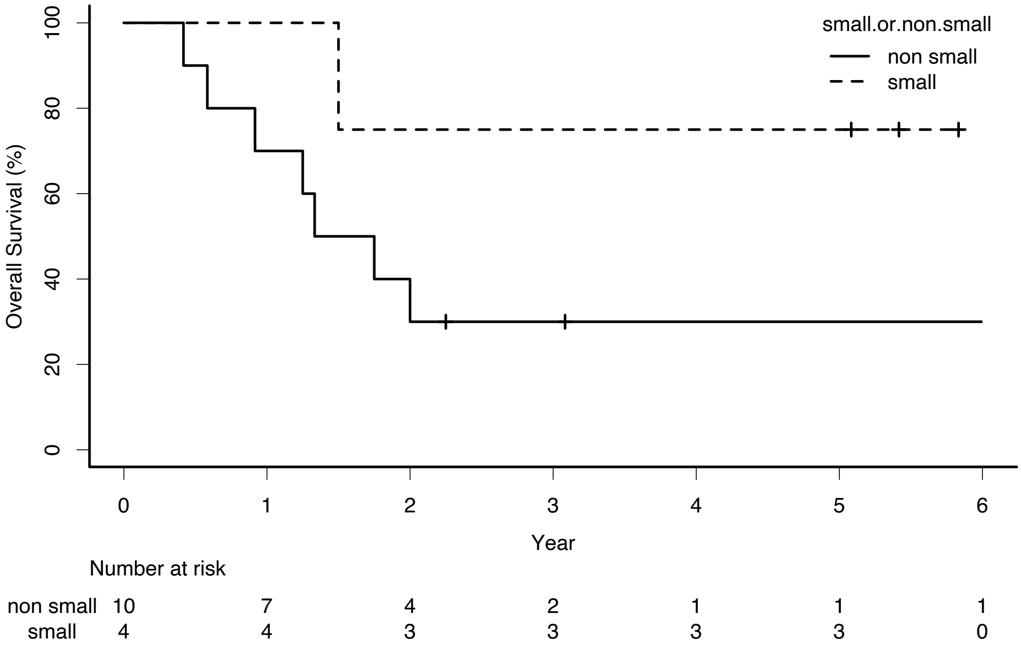

Figure D) Differences in survival according to treatment strategy for non-stage IV (only patients treated with the small cell carcinoma regimen)

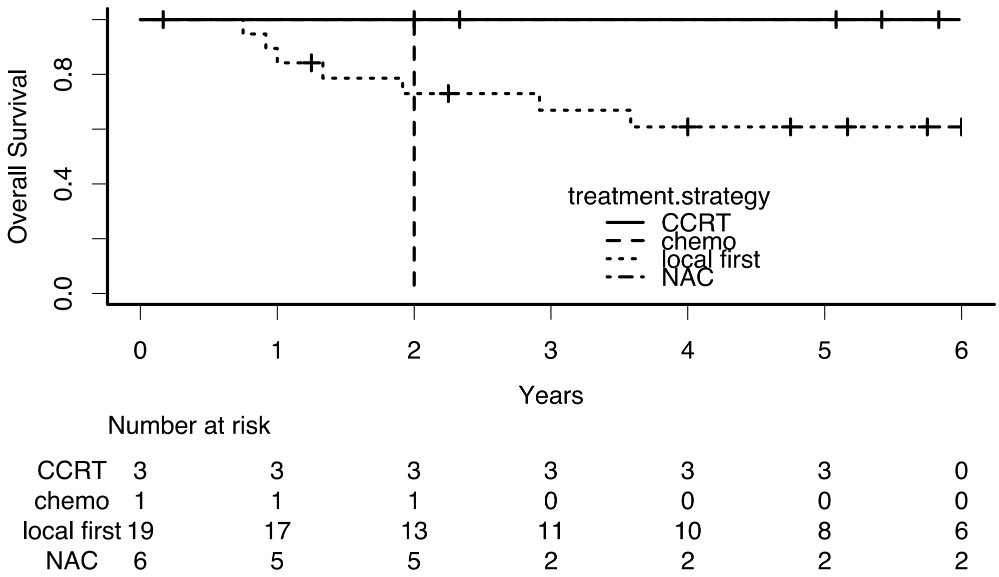

Table E) Characteristics of patients who died from dissemination to the brain and/or meninges.

	Age	Tumor size	FIGO 2018	Treatment strategy	Chemo	Chemotherapy courses
1	51	26 mm	IIIC	Surgery+Chemotherapy	CDDP+VP16	6
2	41	110 mm	IVB	CCRT	wCDDP	unknown
3	24	20 mm	IIIC	Surgery+CCRT	CDDP+VP16	3
4	31	18 mm	IB	Surgery+Chemotherapy	CBDCA+VP16	4
5	62	68 mm	IVB	Chemo	CDDP+VP16	5
6	51	80 mm	IIIC2	Chemo	CDDP+CPT11	8
7	47	66 mm	IVB	Chemo	CDDP+CPT11	6
8	34	54 mm	IIIC2	Chemo	CDDP+CPT11	8
9	64	30 mm	IVB	Chemo	Biweekly CPT11	10
